# Do patients regret having in-office vocal fold injections for glottic insufficiency?

**DOI:** 10.1186/s40463-023-00643-8

**Published:** 2023-04-25

**Authors:** Alice Q. Liu, Yunqi Ji, Amanda Hu

**Affiliations:** 1 Division of Otolaryngology-Head and Neck Surgery, University of British Columbia, Vancouver, BC Canada; 2 Department of Community Health Sciences, University of Calgary, Calgary, AB Canada

**Keywords:** Vocal fold injections, Vocal fold paralysis, Voice handicap index, Patient reported outcomes, Decision regret scale

## Abstract

**Background:**

In-office vocal fold injections (VFI) are an effective treatment for glottic insufficiency. The primary objective of this study was to assess if patients reported decisional regret after VFI. Secondary objectives included determining if variables were associated with lower decisional regret.

**Methods:**

Case–control study of patients who underwent in-office VFIs for glottic insufficiency from August 2017 to December 2019 at a tertiary laryngology clinic. Participants completed the validated Decision Regret Scale (DRS). Demographic data, clinician’s perceptual analysis with GRBAS (Grade, Roughness, Breathiness, Asthenia, Strain), and patient’s self-reported Voice Handicap Index-10 (VHI-10) were analyzed. Nonparametric tests as well as univariate and multiple logistics regression were performed.

**Results:**

Of patients eligible, 75% (136/182) completed the DRS (mean age 65.4 years (SD 13.9), 58.1% male). Eighty-three (61.0%) reported no decisional regret, thirty-three (24.3%) reported mild decisional regret, and twenty (14.7%) reported moderate to strong decisional regret. Improvement in most recent VHI-10 (Kendall correlation coefficient tau = 0.156, *p* = 0.029), Grade of voice (tau = 0.236, *p* value = 0.002) and Breathiness of voice (tau = 0.150, *p* = 0.044) were associated with lower DRS. Multivariate logistics regression results showed that the change in Grade of voice (OR 9.9, *p* < 0.01), Roughness (OR 0.2, *p* < 0.01) and Breathiness (OR 0.2, *p* < 0.03) were significantly associated with DRS.

**Conclusion:**

The majority of patients had no or mild decisional regret after in-office VFI for glottic insufficiency. Both patients who reported less vocal handicap after VFI and clinician-noted improvements in perceptual evaluation of voice after VFI were associated with significantly lower decisional regret.

**Graphical Abstract:**

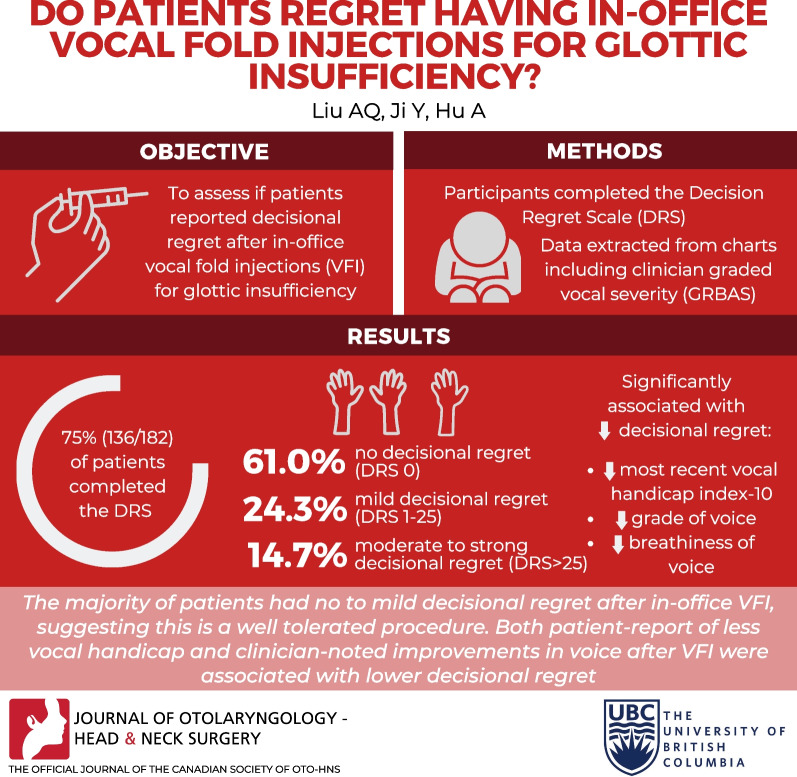

## Background

Vocal fold injections (VFI) are a medialization procedure commonly used in otolaryngology to improve glottic insufficiency. They were first introduced in 1911 and are now commonly completed as  office-based procedures rather than operative procedures requiring general anesthetic [1, 2]. While in-office VFIs have high completion rates with minimal complications and improve voice outcomes [3, 4], there is a paucity of research on patients’ decision-making process with this treatment option.

Increasingly, medicine has focused on patient involvements in the decision-making process and its benefits. Incorporating patients when making decisions reduces decisional conflict, aids in realistic expectations of outcomes, and improves feelings of support [5]. Shared decision making (SDM) is individually tailored and consistent with best practice guidelines [6]. This concept is of particular importance for in-office VFIs as they are performed as an awake procedure, necessitating full cooperation and patient understanding of the procedure to complete it successfully.

A recent review on SDM in otolaryngology discussed integrating patient preferences when there is clinical equipoise or uncertainty [7]. Both of these scenarios can be argued to apply to the treatment of glottic insufficiency. When patients have glottic insufficiency, treatment options include watchful waiting, speech therapy, or elective procedures such as VFI and thyroplasty. There are advantages and disadvantages for each treatment modality, and patients may have similar results with different treatments [4, 8]. Furthermore, patients may be uncertain of whether the vocal fold paralysis that caused the glottic insufficiency will resolve spontaneously. Thus, in-office VFI was felt to be a good example to study SDM in laryngology.

Decisional regret is one aspect of measuring the construct of SDM with patients. Increasing patient understanding of the amount of regret associated with office-based VFIs could improve the discussion providers have regarding informed consent and treatment options for glottic insufficiency. Decisional regret has recently been explored in head and neck oncology and pediatric procedures, but there has been no work in the field of laryngology [9–14].

The primary objective of this study was to assess the amount of decisional regret patients had after in-office VFI for glottic insufficiency. Secondary objectives include analyzing for variables that may be correlated to patient reported decisional regret. We hypothesized that there would be a low level of decisional regret with VFI and that decisional regret would be associated with final voice outcome.

## Methods

This case–control study was approved by the University of British Columbia’s Clinical Research Ethics Board (H20-01747). Inclusion criteria consisted of patients age ≥ 18 years old who were VFI naïve and underwent their first in-office VFI for glottic insufficiency from August 2017 to December 2019 at a single tertiary academic laryngology clinic. Patients were excluded if they were not fluent in English, had poor cognition, had a diagnosis of scar/sulcus vocalis or were deceased at the time of the chart review.

All patients who met inclusion criteria had their charts reviewed, data extracted from each visit in the abovementioned timeframe, and were contacted in regards to their surveys at a later date. Repeat in-office VFIs after first injection were offered for patients in the outlined time frame of this study if there were clinical indications, such as continued aspiration from glottic insufficiency. At our institution, VFIs were completed with hyaluronic acid as an in-office procedure with a previously published technique by a single fellowship trained laryngologist (AH) [4]. VFIs in the operating room or with other materials (e.g. calcium hydroxylapatite) were excluded. VFIs are completed in the operation room only under rare circumstances at our center, such as previous failure of in-office augmentation due to severe anxiety.

The following outcome measurements were routinely recorded at all visits: (1) Auditory perceptual analysis. The Grade, Roughness, Breathiness, Asthenia, Strain scale (GRBAS) allows for perceptual analysis of voice by an expert clinician [15]. Voices were graded on a scale from 0 (normal) to 3 (high degree of abnormality) across five measures.; (2) Patient reported vocal outcomes. The Voice Handicap Index 10 (VHI-10) is a validated 10-item questionnaire measuring patient perceived vocal handicap. Scores range from 0 to 40, with scores greater than 11 considered abnormal. [16] ;(3) Aerodynamic measurement. The Maximum Phonation Time (MPT) is defined as the maximum time a patient can vocalize /i/ after a deep inspiration. Normal values are around 25 s for females and 35 s for males, though this varies with age, stature, and maximal effort [17, 18]. Only data from the initial and most recent visit were used for analysis.

### Decision regret scale

Patients were invited to complete the validated Decision Regret Scale (DRS) via telephone after VFI [19]. The invitation for this survey was mailed out to participants between July to September 2020. The DRS is a simple, five-question survey meant to assess for patient reported decisional regret. Scores range from 0 to 100, with scores ≤ 25 considered mild and scores > 25 indicating moderate to strong decisional regret. The Dillman Total Design Method was used to administer the survey [20]. After mailed invitation letters were sent, scheduled telephone reminders to complete the survey followed at 2-, 4-, and 7-weeks. Data collection closed after 8 weeks. GRBAS was evaluated by the first author (AL) at the time participants completed the DRS. Audio-perceptual evaluation of voice through telemedicine has been shown to be comparable to in-person evaluation [21–23]. There was no financial incentive for patients to participate.

### Data analysis

Descriptive statistics were calculated for all measures. This included the difference in VHI-10 and GRBAS from initial visit, pre-injection, to most recent visit and evaluation.

Since our main outcome measure, DRS, showed a skewed distribution, non-parametric tests were used such as Kendall’s tau correlation test and Mann–Whitney U to identify any significant correlation and association between DRS and potential variables. Assessed variables included age, gender, BMI, duration of hoarseness, professional voice use status, diagnosis (i.e. paralysis, paresis, or presbyphonia), etiology, sidedness, time since injection to survey administration, MPT, GRBAS, and VHI-1. A univariate analysis based on a binary indicator of DRS ≤ 25 (no to mild decisional regret) vs DRS > 25 (moderate to severe decisional regret) was conducted to identify significant association between DRS and those potential variables. A multiple logistic regression included age, gender, change in VHI-10, change in GRBAS, and duration of hoarseness, which were hypothesized to be possible contributors to DRS. Variables were entered in a backward stepwise regression technique. Age and gender were always included in each step of model selection process. A receiver operating characteristic curve was run to confirm the diagnostic accuracy of the fitted model. In addition, subgroup analysis with an unpaired Student’s t-test or Mann Whitney U test was completed to compare the mean measurements of those with no to mild decisional regret (DRS ≤ 25) to those who had moderate to strong decisional regret (DRS > 25). Fisher’s exact test was used to analyze if there was a difference between the proportion of patients who subsequently underwent thyroplasty in the no to mild decisional regret (DRS ≤ 25) group versus the moderate to strong decisional regret (DRS > 25) group. All analyses were done using *R 4.0.5* software and a priori significance was defined as *P value* < *0.05.*

## Results

### Patient information

**Fig. 1:**
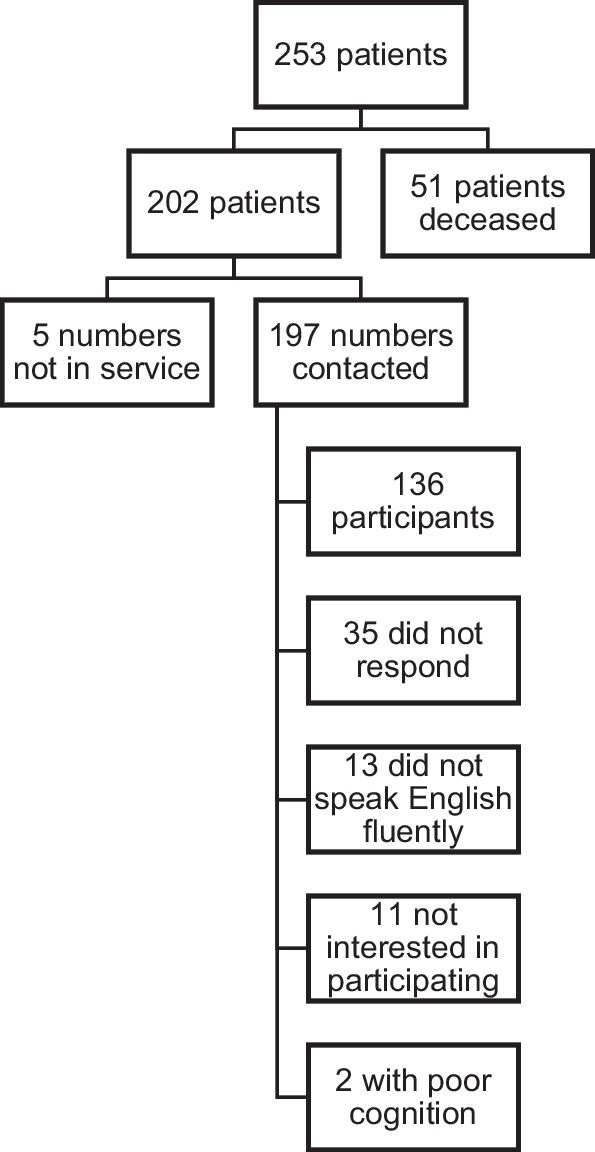
Attrition and response rate of patients who were contacted to complete the Decision Regret Scale and GRBAS from August 2017 to December 2019

In total, 253 consecutive patients underwent VFIs between August 2017 to December 2019. Patients were excluded from participating in the DRS for the following reasons: 51 patients were deceased, 13 patients did not speak English, 5 patients’ telephone numbers were not in service, and 2 patients had poor cognition. This left 182 eligible patients. With initial contact between July to September 2020, 74.7% of eligible patients (136/182) completed the DRS (mean age 65.4 years (SD 13.9), 58.1% male). Thirty-five (19.2%) patients did not respond and 11 (6.0%) patients declined participation. The attrition and response rate of patients are shown in Fig. 1. Patients who completed the survey were not significantly different than eligible patients who did not participate in terms of age, gender, or etiology of VFI. The majority of patients had vocal fold paralysis (52.2%), followed by vocal fold paresis (24.3%), and presbyphonia (23.5%). Full demographic data of the 136 patients who completed the DRS is shown in Table 1. Sixteen (11.8%) patients who partook in the DRS went on to have a thyroplasty during the follow-up period of this study.

**Table 1:** Demographic data of included patients, on first baseline visit, who underwent in-office vocal fold injections of hyaluronic acid between August 2017 to December 2019 (*n* = 136)

Variable	Data
*Gender, n (%)*	
Female	57 (41.9%)
Male	79 (58.1%)
*Diagnosis, n (%)*	
Paralysis	71 (52.2%)
Paresis	33 (24.3%)
Presbyphonia	32 (23.5%)
*Etiology, n (%)*	
Iatrogenic	55 (40.4%)
Idiopathic	50 (36.8%)
Malignant	17 (12.5%)
Neurological	7 (5.2%)
Other	7 (5.2%)
*Side of vocal fold pathology, n (%)*	
Left	71 (52.2%)
Right	30 (22.1%)
Bilateral	35 (25.7%)
*Professional voice user, n (%)*	
Yes	29 (21.3%)
No	107 (78.7%)
*Age (years)*	
Mean (SD)	65.4 (13.9)
*Duration of hoarseness (months)*	
Mean (SD)	15.5 (30.7)
*BMI*	
Mean (SD)	26.2 (7.7)
*Voice Handicap Index-10 (baseline)*	
Mean (SD)	24.0 (9.1)
*GRBAS–Grade on baseline visit*	
0	0.8%
1	16%
2	47.2%
3	36%
*Maximum Phonation Time (seconds)*	
Mean (SD)	7.20 (5.47)
Subsequent thyroplasty, n (%)	16 (11.8%)
*Time from in-office vocal fold injection to survey administration (months)*	
Mean (SD), range	20,1 (8.1), 8.3–35.7

### DRS

**Fig. 2:**
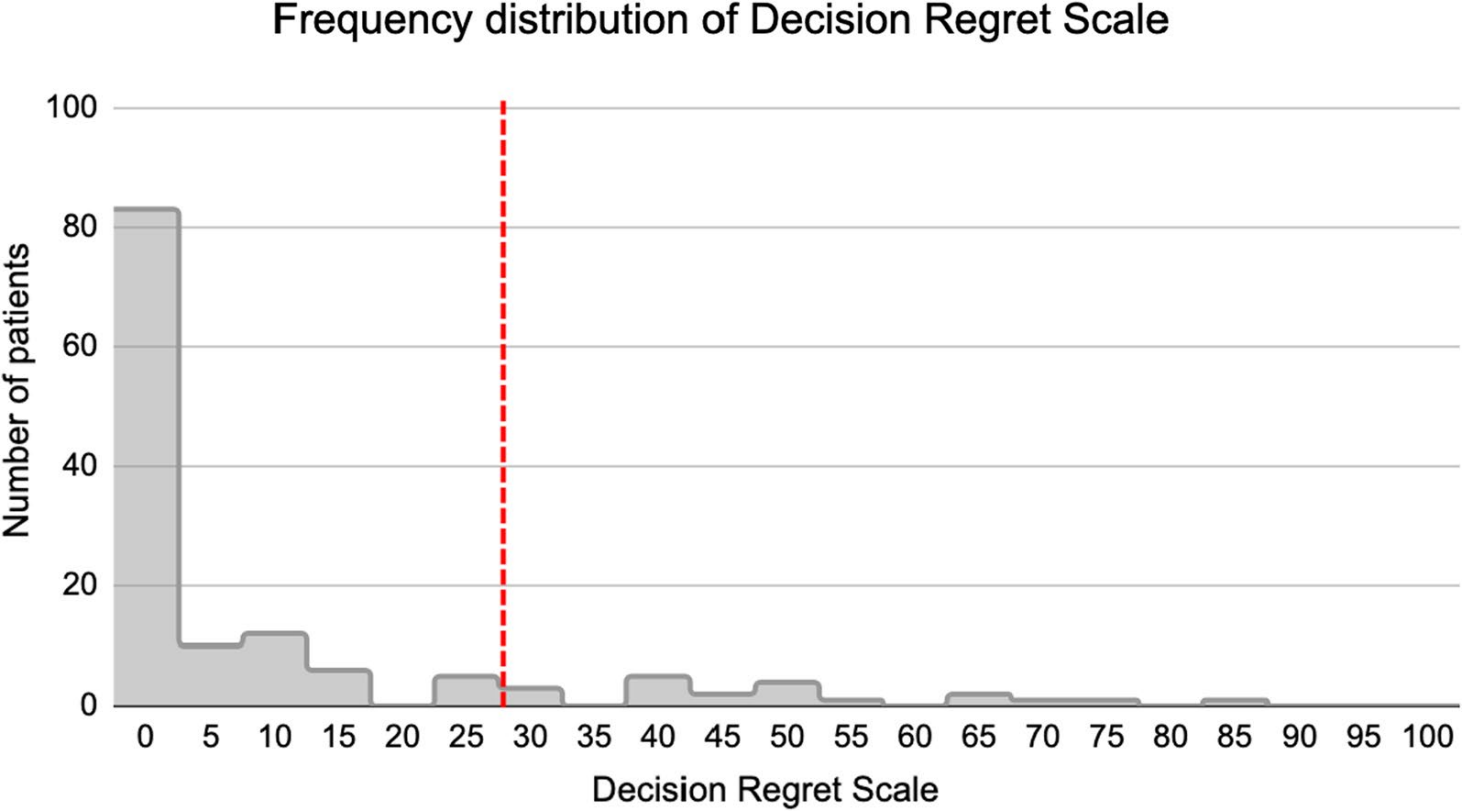
Frequency distribution of patients’ Decision Regret Scale scores (*n* = 136). Vertical line separates scores ≤ 25 (no to mild decisional regret) from scores > 25 (moderate to severe decisional regret)

Eighty-three (61.0%) participants reported no decisional regret, thirty-three (24.3%) reported mild decisional regret, and twenty (14.7%) reported moderate to strong decisional regret. Figure 2 shows a skewed distribution of reported DRS scores towards lower scores (i.e. no or mild decisional regret). The median DRS was 0 (IQR 0–10), while the mean DRS for participants was 10.14 (SD18.4). Table 2 displays the score for each item on the DRS. “I would make the same choice again” was the item that prompted the greatest decisional regret (median 0 (IQR), mean 15.7 (SD31.66)).

**Table 2:** Overall Decision Regret Scale (DRS) inventory

Question	Mean (SD)*	Median (IQR)*
1. It was the right decision	9.74 (21.77)	0 (0)
2. I regret the choice that was made	6.61 (20.69)	0 (0)
3. I would make the same choice again	15.07 (31.66)	0 (0)
4. The choice did me a lot of harm	8.27 (20.42)	0 (0)
5. The decision was a wise one	11.03 (23.13)	0 (6.25)
Total decisional regret	10.15 (18.44)	0 (10)

*Scores range from 0 to 100, with higher scores indicating greater regret

### VHI-10

**Fig. 3:**
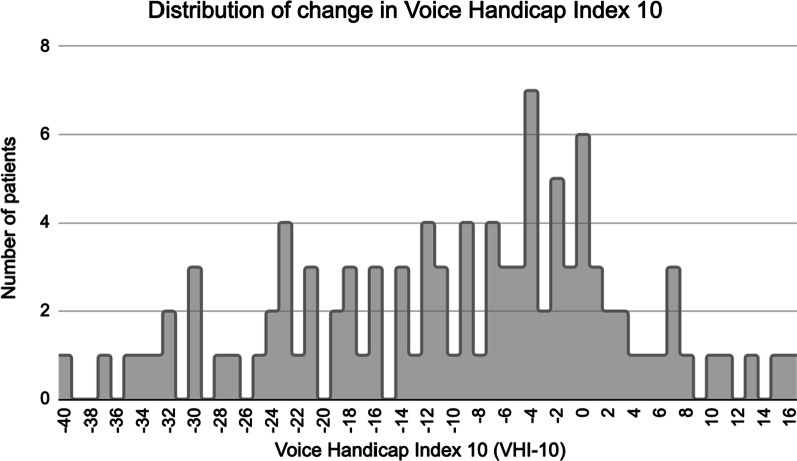
Frequency distribution of included patients and their change in Voice Handicap Index 10 (VHI-10). The minimal clinically important difference of VHI-10 is 4 from a previously published study on vocal fold paralysis

At baseline, patients started with an abnormal mean VHI-10 score of 24.0 (SD 9.1) The difference in VHI-10 scores showed that the majority of patients reported improved voice outcomes over time after VFIs. Figure 3 displays the frequency distribution of change in VHI-10 scores.

### Statistical analysis

**Fig. 4:**
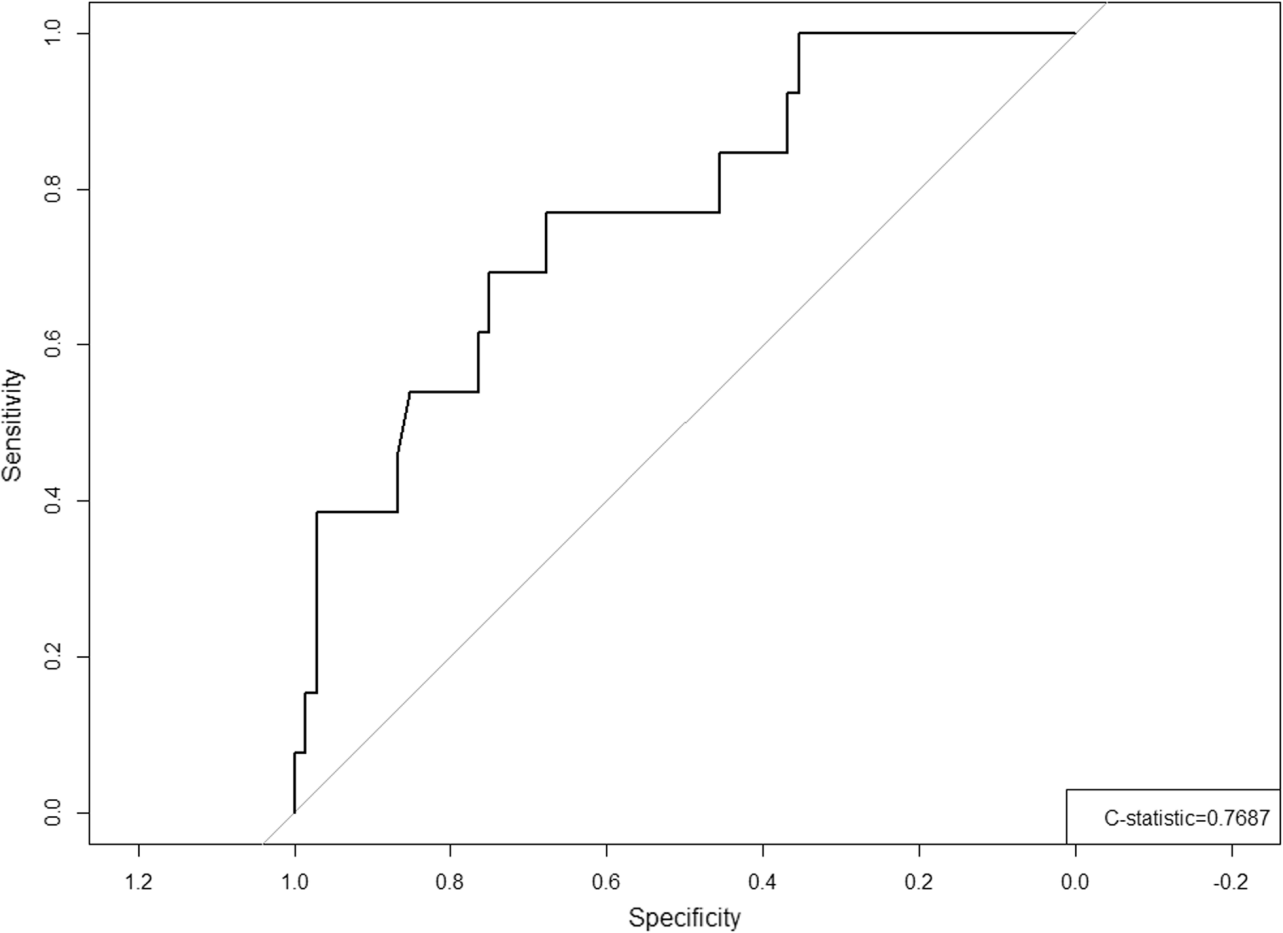
The Receiver Operating Characteristic (ROC) Curve of the Fitted Model

DRS had no correlation with demographic factors, diagnosis, or time since the injection (mean 20.1 months [SD 8.1 months]). The results of the univariate regression did not find any significant variables. Kendall correlation analysis reported that improvements in VHI-10 scores (tau = 0.156, *p* = 0.029), Grade of voice (tau = 0.236, *p* value = 0.002), and Breathiness of voice (tau = 0.150, *p* = 0.044) compared to baseline values were associated with lower DRS (Table 3). There was no association between DRS and change in MPT from baseline. The results of the multiple logistics regression model reported that a change in Grade of voice at time of survey administration, (OR 5.070, *p* value < 0.01), change in Roughness (OR 0.366, *p* value < 0.05) and change in Breathiness (OR 0.326, *p* < 0.05) were predictors for DRS. Refer to Table 4 for full results of the multiple logistic regression. Figure 4 shows the c-statistics of the receiver operating curve is 0.769, confirming diagnostic accuracy of the fitted model.

**Table 3:** Correlation analysis between potential variables and decision regret scale

	Mann Whitney U Test for categorical variables / Kendall Correlation Tau for continuous variables	*P*-value
Gender	2602	0.079
Professional voice user	1485	0.690
Diagnosis	0.110	0.946
Etiology	7.039	0.134
Side of vocal fold pathology	0.376	0.829
Age	0.103	0.115
Most Recent VHI	0.156	0.029*
Change in VHI	0.090	0.275
Change in GRBAS – Grade	0.236	0.002*
Change in GRBAS – Roughness	− 0.049	0.529
Change in GRBAS –Breathiness	0.150	0.044*
Change in GRBAS –Asthenia	0.117	0.130
Change in GRBAS –Strain	0.067	0.392
BMI	0.031	0.811
Change in Maximum Phonation Time	0.285	0.046
Duration of hoarseness (months)	0.035	0.605

*Denoting significance *P* < 0.05

**Table 4:** Multiple logistic regression model of selected variables and Decision Regret Scale (DRS)

Variable	Odds ratio	95% confidence interval Lower bounds	95% confidence interval Upper bounds	Two-tailed *p*-value
Gender	0.683	0.177	2.630	0.579
Age	0.900	0.944	1.038	0.666
Change in Grade	5.070	1.539	16.700	0.008*
Change in Roughness	0.366	0.160	0.835	0.017*
Change in Breathiness	0.326	0.127	0.839266	0.021*

*Denoting significance *P* < 0.05

Table 5 presents the subgroup analysis of patients who had no to mild decisional regret (DRS ≤ 25) versus patients who had moderate to strong decisional regret (DRS > 25). The difference in VHI-10 from initial visit to most recent was less when comparing those with moderate to strong decisional regret to those with no to mild decisional regret (mean change of − 8.4 vs mean change of − 9.8). The absolute value for the most recent VHI-10 was also higher in those who had moderate to strong decisional regret (18.4 vs. 15.6). However, these findings were not significant (*p* = 0.664 and *p* = 0.417 respectively). There was also no association between DRS and patients who subsequently underwent thyroplasty surgery on Fisher’s exact test (*p* = 0.277).

**Table 5:** Comparison of patient outcomes for those who scored ≤ 25 on the Decision Regret Scale (DRS) compared to those who scored > 25

	Decision Regret Scale ≤ 25 No to mild decisional regret	Decision Regret Scale > 25 Moderate to strong decisional regret	*P*-value*
N	116 (85.3%)	20 (14.7%)	
Age (mean) (SD)	65 (14.1)	68 (13.0)	0.375
Most recent VHI-10 (mean) (SD)	15.6 (10.3)	18.4 (11.9)	0.416
Change in VHI-10 from baseline to most recent (mean) (SD)	− 9.9 (12.5)	− 8.4 (13.2)	0.664
Change in Grade from baseline to most recent (mean) (SD)	− 1.67 (1.10)	− 1.26 (0.87)	0.083
Change in Roughness from baseline to most recent (mean) (SD)	0.04 (0.98)	− 0.32 (0.95)	0.148
Change in Breathiness from baseline to most recent (mean) (SD)	− 1.46 (1.27)	− 1.21 (1.40)	0.471
Change in Asthenia from baseline to most recent (mean) (SD)	− 1.13 (0.91)	− 1.05 (0.85)	0.713
Change in Strain from baseline to most recent (mean) (SD)	− 0.25 (0.73)	− 0.05 (1.03)	0420
Duration of hoarseness in months (median) (IQR)**	4 (9.5)	66(10.5)	0.462
Subsequent thyroplasty, n (%) ***	15 (11.0%)	1 (0.7%)	0.277

None of the variables were statistically different between the two groups

* P-values were calculated with two-tailed Students t test

**The Median and IQR were presented as the data were skewed and P-values were calculated with Mann Whitney U Test

*** Fisher’s exact test was used as proportions were compared

## Discussion

To our knowledge, this is the first study exploring patient reported decisional regret in laryngology. Glottic insufficiency can impact a patient’s quality of life in many ways, including dysphonia, aspiration risks, or airway compromise [24]. VFIs are often used as a safe, temporizing treatment for glottic insufficiency to improve voice outcomes [2–4]. Patient satisfaction with VFIs are reflected in the low DRS scores reported in this study; the majority of our patients reported no (61.0%) or mild (24.3%) decisional regret (Fig. 2). A systematic review of 59 studies using the DRS found the mean score was 16.5, higher than this study’s mean score of 10.15 [25]. In-office VFIs appear to cause less decisional regret than many other healthcare interventions, suggesting this is a well tolerated procedure.

When looking at variables associated with decisional regret with VFIs, patient-reported improvement in vocal handicap and clinician’s perceptual analysis of voice were significantly associated with decisional regret on Kendall correlation analysis. The multiple logistic regression had three attributes of perceptual analysis of voice as significant predictors for decisional regret: change in Grade, change in Roughness, and change in Breathiness (Table 3). However, VHI-10 was not significant on multivariate analysis with DRS. Nevertheless, there was a notable trend in decreasing VHI-10 from initial visit to most recent visit after VFI. The mean change in VHI-10 was -9.61 across all included patients, with larger decreases in patients who had no to mild decisional regret (Table 4). The minimal clinically important difference (MCID) of VHI-10 has been previously reported as 4 in a study of vocal fold paralysis patients, which the majority of our patients did meet (Fig. 3) [26]. However, the authors acknowledge that the MCID may vary depending on the situation and intervention used.

Our results showed that demographic variables, diagnostic variables, or having a thyroplasty did not significantly correlate to DRS. Patient reported outcomes and clinician noted improvements in voice were the only variables related to DRS. Our high survey response rate of 74.7% is similar to other studies using the Dillman Total Design Method in otolaryngology [27–29], and can be used as a surrogate marker for the validity of our results [21–30]. Importantly, we found no significant correlation between time since injection to survey and DRS. Hyaluronic acid, the injection material used for this procedure, takes 4–6 months to resorb [32]. Therefore, only patients who had at least this amount of time since first injection were included in our patient population to allow patients to experience the full effect of the temporary injection.

The DRS has been used in other fields of otolaryngology, including pediatrics and head and neck oncology. In Hong et al.’s study on pediatric adenotonsillectomy or tonsillectomy, 54.7% of parents reported no regret, 43.7% reported mild regret, and only 1.6% reported moderate to strong decisional regret [10]. The only demographic variable that had difference in total DRS score was postoperative complications. This study shows less moderate to strong decisional regret for an elective otolaryngology procedure than ours, but it is difficult to directly compare as parents were reporting these results for their children.

In head and neck oncology, 15.5–26.7% of patients reported moderate to severe decisional regret [9, 33]. This was slightly higher than the 14.7% reported in our study. Major head and neck oncological procedures are more invasive and carry higher risks of complications than in-office VFIs. Similar to our study, age was not related to DRS in a study of patients undergoing major head and neck procedures [9]. Interestingly, preoperative depression was the only factor associated with moderate to severe decision regret.

Shuman et al. examined decisional regret in laryngeal cancer specifically [12]. They also found that patients who reported worse vocal quality of life measures experienced more decisional regret (*p* value < 0.001). Even though laryngeal cancer is a diagnosis that threatens survival, poor voice outcomes were significantly associated with decisional regret. In a tradeoff between survival or speech, the Fireman study found that 20% of patients would choose a 30% reduction in survival to avoid laryngectomy and preserve near normal speech [34]. This illustrates the importance of the human voice to patients. As healthcare providers, these findings emphasize the critical choices patients make for quality of life and survival. Table 6 summarizes the amount of decisional regret after procedures in various otolaryngology subspecialties.

**Table 6:** A comparison of the distribution of Decisional Regret Scale scores after various procedures

Authors	Indication or procedure	Subspecialty	No decisional regret (DRS 0)	Mild decisional regret (DRS 0 ≤ 25)	Moderate to severe decisional regret (DRS > 25)
Current study	Adult in-office vocal fold injections	Laryngology	61.0%	24.3%	14.7%
Hong P, Maguire E, Purcell M, Ritchie KC, Chorney J	Pediatric adenotonsillectomy or tympanostomy	Pediatrics	54.7%	43.7%	1.6%
Hong P, Gorodzinsky AY, Taylor BA, Chorney JM	Pediatric otoplasty	Pediatrics	59.7%	35.5%	3.2%
Thomas CM, Sklar MC, Su J, et al	Major head and neck procedure	Head & Neck Oncology	36.7%	36.7%	26.7%
Goepfert RP, Fuller CD, Gunn GB, et al.	Oropharyngeal squamous cell cancer	Head & Neck Oncology	38.6%	45.8%	15.5%

Pediatric procedures had the DRS scored by patients’ caregivers

Clinical ramifications of this work include clinicians being aware that there was minimal regret after VFIs for glottic insufficiency. As shown in Table 6, VFIs had the highest percentage of no decisional regret compared to the other four studies in otolaryngology. The largest amount of regret was noted with Q3 on the DRS, “I would go for the same choice again” (Mean score 15.07) (Table 2). This is contrasted to the study examining patients who underwent treatments for oropharyngeal cancer, which had Q4 “The choice did me a lot of harm” as their greatest regret (Mean score 27.0) [33]. Perhaps this supports the fact that patients do acknowledge multiple treatment options are available, and some regret not exploring options such as watchful waiting to see if vocal fold function would recover.

The continued analysis of decisional regret in otolaryngology, as well as decision making, has large implications on ways to improve patient care. When patients are involved in the decision-making process, and have an informed view of treatment options, it is well supported that they also have less regret [35, 36]. Future work could compare decisional regret in VFIs to other treatment modalities for glottic insufficiency, such as thyroplasty or watchful waiting.

Limitations for this study includes inherent self-selection bias as we were only able to analyze data from those who chose to participate. Since this was a retrospective survey, patients may also have recall bias. Patients who were deceased, unable to be reached, or could not speak English were excluded due to practical reasons. It is plausible that their responses may have changed the findings. Unexpectedly, a surprising finding in our patients was that 20.2% (51/253) had expired within the follow-up period of this study (Fig. 1). These patients passed away from reasons unrelated to their VFIs, such as having terminal malignancies or recent major cardiac procedures. VFIs may have improved the quality of life of these patients, but this would not have been captured due to the high mortality rate. Due to COVID-19 precautions, we could also not perform this study in person at set follow-up times. Telephone delivery was chosen as it still allowed us to perform audio-perceptual analysis, minimized in-person contact, and has the highest response rate of survey delivery methods [31]. Previous studies have demonstrated that audio-perceptual evaluation of voice is comparable for in-person evaluations and telemedicine [21–23]. However, we were not able to include laryngoscopy or evaluate the size of the glottic gap as outcome measures. Furthermore, the costs associated with VFIs are covered by the provincial government at our clinic due to its location in a tertiary academic hospital. Therefore, the cost of injectable materials was not assessed in patients’ decisional regret scores. It is possible that patients could have undergone other procedures between the timing of VFI and DRS that could influence the results (e.g. thyroidectomy, lung surgery, etc.). There is, however, a provincial computer system that would have captured the majority of these in-hospital procedures. Finally, this study examined a single provider’s practice, and clinician-patient interaction likely impacts decisional regret. We acknowledge these results may not be as generalizable to other settings.

## Conclusion

This is the first study to examine decisional regret in laryngology. We discovered that, from a patient perspective, the majority of patients had no or mild decisional regret after VFIs. Improvement in self-reported vocal handicap and perceptual analysis of voice by a skilled clinician were significantly correlated with decisional regret. The results of this study will help improve the informed consent process and management of patient expectations regarding this common procedure in otolaryngology.

## Data Availability

The datasets used and/or analysed during the current study are available from the corresponding author on reasonable request.

## Abbreviations

VFI: Vocal fold injection
SDM: Substitute decision making
DRS: Decision regret scale
GRBAS: Grade, roughness, breathiness, asthenia, strain scale
VHI-10: Vocal handicap index-10
MPT: Maximum phonation time
MCID: Minimal clinically important difference
